# Optimizing Tobacco Advertising Bans in Seven Latin American Countries: Microsimulation Modeling of Health and Financial Impact to Inform Evidence-Based Policy

**DOI:** 10.3390/ijerph18105078

**Published:** 2021-05-11

**Authors:** Ariel Bardach, Andrea Alcaraz, Javier Roberti, Agustín Ciapponi, Federico Augustovski, Andrés Pichon-Riviere

**Affiliations:** 1 Department of Health Technology Assessment and Economic Evaluation, Institute for Clinical Effectiveness and Health Policy, Ravignani 2024, Buenos Aires 1414, Argentina; abardach@iecs.org.ar (A.B.); aalcaraz@iecs.org.ar (A.A.); aciapponi@iecs.org.ar (A.C.); faugustovski@iecs.org.ar (F.A.); apichon@iecs.org.ar (A.P.-R.); 2 CIESP–CONICET, Centre for Research in Epidemiology and Public Health/National Scientific and Technical Research Council, Ravignani 2024, Buenos Aires 1414, Argentina

**Keywords:** tobacco, smoking, advertising and sponsorship ban, financial benefits, health benefits, Latin America

## Abstract

Introduction: In Latin America, tobacco smoking prevalence is between 6.4% and 35.2%. Governments have been making efforts to support the regulation of advertising and, in many cases, banning advertising and promotion of tobacco altogether. The objective of this study was to evaluate the potential impact on health and economic outcomes of optimizing a ban on tobacco advertising and sponsorship in Argentina, Bolivia, Brazil, Chile, Colombia, Mexico, and Peru. Methods: We built a probabilistic microsimulation model, considering natural history, direct health system costs, and quality of life impairment associated with main tobacco-related diseases. We followed individuals in hypothetical cohorts and calculated health outcomes on an annual basis to obtain aggregated 10-year population health outcomes (deaths, events, healthy years of life) and costs. To populate the model, we performed a rapid review of literature to calculate intervention effectiveness. Results: With current policies, over 10 years, in Argentina, Bolivia, Brazil, Chile, and Colombia a total of 50,000 deaths and 364,000 disease events will be averted, saving $7.2 billion. If the seven countries strengthened their policies and implemented a comprehensive ban with 100% compliance, 98,000 deaths and 648,000 events would be averted over 10 years, saving almost $15 billion in healthcare costs. Conclusions: Optimizing a ban on tobacco advertising and sponsorship would substantially reduce deaths, diseases, and health care costs attributed to smoking. Latin American countries should not delay the full implementation of this strategy.

## 1. Introduction

Every year, over seven million people die around the world because of the tobacco pandemic, the single most preventable cause of premature mortality; moreover, between 1990 and 2017, the total number of smoking-attributable deaths increased by 24.9% [1]. The expenditure in healthcare for smoking-attributable diseases currently exceeds USD 400 billion per year and the economic cost of smoking represents USD 1436 billion [2]. In Latin America, the annual consumption of tobacco per person ranges from 160 to 2000 cigarettes and the tobacco smoking prevalence between 6.4% and 35.2% [3]. 

Tobacco advertising in its many forms increases tobacco consumption [4]. Marketing of tobacco products includes direct advertising on television, radio, magazines, newspapers, billboards, and retail points-of-sale (POS) whereas indirect marketing refers to the free distribution of products, promotional discounts, the appearance of tobacco products in television or films, sponsorship of sport and music events, and the distribution of nontobacco products identified with tobacco brand names [5]. Since the early days of the entertainment industry, audiences have viewed smoking in films; and the contribution of this strategy in the promotion of smoking has been long studied, with a focus on smoking initiation in adolescents [6,7,8,9,10]. Indeed, POS marketing has been associated with an increased smoking susceptibility, experimentation, and uptake [11,12,13]. A more recent and growing marketing strategy is the tobacco advertising and branding on social media and other internet-based advertising [14,15]. 

Governments have been making efforts to strengthen the regulation of advertising and, in many cases, banning it altogether [4]. In fact, although comprehensive bans of advertising can result in a decline in the per capita consumption, limited bans have no significant impact on consumption because it leads to a change of advertising media, from those banned media towards those media that are still allowed [4,16]. On the opposite side, tobacco and advertising industries contend that these marketing regulations have negative economic consequences, infringe legal rights of tobacco companies, create regulatory redundancy, and that evidence supporting their effects is insufficient [17]. 

In 2008, the member states of the World Health Organization (WHO) adopted a treaty to provide a context for the development of tobacco control policy; the WHO Framework Convention on Tobacco Control (FCTC). The FCTC introduced a practical and cost-effective package of six evidence-based measures, referred to as MPOWER, and these measures correspond to the provisions of the framework. These measures include: Monitoring tobacco use and tobacco control measures (Article 20); Protecting people from tobacco smoke (Article 8); Offering help to quit tobacco (Article 14); Warning people about the dangers of tobacco (Articles 11 and 12); Enforcing bans on tobacco advertising, promotion and sponsorship (Article 13); and Raising tobacco taxes (Article 6) [18,19]. Despite an accelerating impact of MPOWER policies, the implementation of legislation related to tobacco advertising at a global level is still limited [20,21,22]; moreover, scarcity of quality information at a country-level, lobbying, and constituency building from the tobacco industry have delayed the implementation and enforcement of these measures in Latin America [23,24]. In the group of studied countries, Peru and Bolivia have the minimum level of implementation; Argentina and Mexico are in an intermediate level of implementation with a ban of advertising on national television, radio, print media, and some direct or indirect forms of advertising, and although Colombia, Brazil, and Chile have the maximum level of implementation with a comprehensive ban of all forms of advertising, there is still room for improvement in the compliance of measures [25]. 

The objective of this study was to assess the 10-year health and economic impact of the current country-specific legislation and implementation related to bans on tobacco advertising, promotion, and sponsorship; and to compare this impact to the expected effects of moving to total ban of advertising in Argentina, Bolivia, Brazil, Chile, Colombia, Mexico, and Peru, using a probabilistic state-transition microsimulation model developed to estimate the burden of smoking-attributable disease and the cost-effectiveness of tobacco control policies and interventions in Latin America.

## 2. Methods

The model used in this study is an individual-based Markov model, or first-order Monte Carlo technique [26]. The smoking burden model allows for the estimation of health and economic impact of tobacco, at present and after implementation of interventions to reduce smoking prevalence. This model has been validated and applied in studies carried out in 12 Latin American countries that estimated the burden of disease and the expected impact of tobacco tax increases and other strategies [25,27,28,29,30,31,32,33]. 

### 2.1. Disease Burden Modeling

The model considers the natural history, the costs, and the quality-of-life losses associated with main tobacco-related diseases (coronary and non-coronary heart disease, cerebrovascular disease, chronic obstructive pulmonary disease, pneumonia, influenza, lung cancer, and nine other neoplasms). We followed up individuals in hypothetical cohorts and calculated health outcomes on an annual basis to obtain aggregated long-term population health outcomes and costs. For acute events, we calculated age and gender-specific absolute risks based on national mortality rates and the lethality of the event. Then, the baseline risk in non-smokers is calculated based on the smoking prevalence in each age and sex group, and the relative risk of smoking associated with each event. For cancers, we obtained incidence statistics for each age and sex with Global Cancer Observatory (GCO -GLOBOCAN) for each country [34].

The model updates the input parameters for each subject in yearly cycles and calculates individual lifetime risks of occurrence of each event, disease progression, and death, based on demographic attributes, smoking status, and clinical conditions according to the underlying risk equations. The main outcomes are life years, quality-adjusted life years, disease events, hospitalizations, disease incidence, and disease costs. We calculated the years of life lost (YLL) due to smoking-related diseases at a population level as the sum of years of life lost due to premature death (PYLL); and years of life lost due to living with a poor quality of life (YLL-QL). As the model does not directly calculate the consequences of passive smoking and perinatal effects, based on the results of previous studies, we assumed that these causes impose an additional burden of 13.6% for men and 12% for women [35].

### 2.2. Policy Effectiveness Modeling

Tobacco control policies have an effect mediated by a reduction in consumption. This lower consumption at the country level is a consequence of both a reduction in the number of cigarettes smoked per smoker, and lower tobacco prevalence due to an increase in quitting rates (short term) and lower tobacco initiation rates in the medium and long term. To estimate the impact of implementing strategies on tobacco advertising and sponsorship ban, the smoking prevalence post-intervention was calculated as:

*Prevalence_post_* = *Prevalence_pre_* − (*Em* ∗ *Ip* ∗ *Prevalence_pre_*)

where *Prevalence_pre_* is the prevalence of smokers before the intervention, *Em* is the effectiveness of the intervention expressed as relative reduction in tobacco consumption, and *Ip* it is the proportion of variation in consumption that impacts smoker prevalence. Different studies have estimated that, in the short and medium term, approximately half of the reduction in consumption is a consequence of reduced prevalence and the other half is explained by reduced consumption of continuing smokers [36,37,38,39,40]. 

### 2.3. Model Scenarios and Base Case Analysis

To estimate the potential impact of banning advertising, promotion, and sponsorship, we considered and analyzed progressive estimates, over ten years, in each country in three scenarios (short-term: 2 years, mid-term: 5 years, and long-term: 10 years) to calculate the reduction of the health burden associated with the reduction in cigarette consumption.

1. Short-term scenario: In this scenario, we assumed that a 50% reduction in consumption would have an impact on prevalence (Ip = 0.5) which, in turn, led to an increase in former smokers. This conservative scenario is more likely to occur in the short term, as it does not include the intervention effects in preventing people from starting to smoke or the health benefits of smoking fewer cigarettes for those who continued smoking.
2. Mid-term scenario: Similar to the previous scenario, but also including the potential effects associated with a decrease in number of cigarettes smoked in people who continued smoking. Considering that low-intensity smokers have, on average, 75% less excess disease risk than a high-intensity smoker when compared to non-smokers (82% less for lung cancer, 57% less for ischemic heart disease and 80% less for chronic obstructive pulmonary disease (COPD)), we assumed that a reduction in the number of cigarettes would result in a proportional reduction in the 75% of the excess risk difference between a smoker and a former-smoker [41].
3. Long-term scenario: This is the maximum effect over ten years. Similar to the previous scenario but with a 75% reduction in consumption affecting prevalence (Ip = 0.75); the population of former smokers remains constant in relation to the baseline, with decrease in prevalence and an increase in the number of non-smokers.

The base case consisted of comparing health benefits and costs of current tobacco advertising and sponsorship policy in each country to those predicted by implementing a complete ban. To estimate disease burden and costs of the strategy, we assumed a lineal evolution from scenario 1 to scenario 2 within five years, and then to scenario 3 between years six to ten. 

The burden of disease attributable to smoking was estimated for these scenarios based on these estimates of changes in smoking prevalence and new proportions of smokers, former smokers, and non-smokers. Health impact was calculated as the observed difference between baseline burden (status quo) and the complete ban strategy estimates, in terms of deaths, disease events, years lived, disability, and health costs. More information about the model can be found in the publications in which it was described, evaluated, or used [25,26,27,28,29,30,31,32,33,42,43,44,45], and in the technical reports with findings on the burden of disease (available from www.iecs.org.ar/tabaco, access date 30 March 2021). 

### 2.4. Information Sources for the Model

#### Epidemiological Information

To populate the simulation model, we obtained data through a review of the literature on MEDLINE, EMBASE, CENTRAL, SOCINDEX, EconLit, LILACS, NBER, CRD and Cost Effectiveness Analysis Registry, the International Tobacco Health Conference Paper Index, and Cochrane Tobacco Addiction Review Group register. In addition, we reviewed grey literature from ministries of health or of finance, Pan American Health Organization, and regional congresses proceedings. We obtained updated information on tobacco use from Global Adult Tobacco Surveys (GATS) and national risk factor surveys. Researchers from participating countries provided information from civil registrations, vital statistics, and hospital discharge databases to estimate specific case fatality rates. 

### 2.5. Cost Information

We performed a literature search to identify reported costs of events and developed a common costing methodology to estimate costs through a micro-costing or macro-costing approach, depending on the information availability. Then, we used a spreadsheet for each event, with the frequency, the use rate, and the unit cost of health resources. We constructed ad hoc micro-costing exercises, based on experts’ opinions, clinical guidelines, and a review of healthcare facility records. The costs of malignancies other than lung cancer were based on cost of each cancer relative to lung cancer costs and consensus using a Delphi method exercise with oncology experts from studied countries. Where local information was unavailable, we extrapolated the model to approximate costs of events. In those cases, we used the average proportion that represents event cost divided by per capita GDP in Argentina, Chile, and Mexico; then, on this average proportion, the per capita GDP of the country of interest was applied to obtain estimates. 

All costs were first estimated in the local currency; consumer price indices, published by the statistics institutes of each country, were used for cost adjustments. Finally, for comparability, all costs in local currency were converted to International dollar (I$) based on the World Bank purchasing power parity exchange rates for 2018. 

### 2.6. Estimation for Intervention Impact

For the assessment of the impact of banning tobacco advertising, promotion, and sponsorship, three possible levels of implementation were considered. At the minimum level of implementation, the absence of a ban and a ban restricted only to national television, radio, or print media were included. The literature agrees that this type of ban has limited or no effect; so, we treated this as absence of a ban. This minimum or no implementation is used as the reference category, with no effectiveness (0%). The intermediate level of implementation covers national television, radio, or print media, and some direct and/or indirect forms of advertising. Our review showed that the effectiveness on relative reduction in per capita consumption is wide-ranging, from 0% to 13.6%; we selected 1% as central estimator as it was the most consistent value suggested by the major sources and used in a study including 102 countries. The maximum level of implementation was the ban of all forms of advertising, direct and indirect, including product display at POS. In this category associated with legislation, the level of compliance to the ban will clearly determine effectiveness and the potential impact of taking the measure to the maximum level. The range of reduction in per capita tobacco consumption associated with this level is 5% to 23.5% (see Table 1). As a central estimator, the two best options were: a reduction of cigarette consumption of 7.4% described for 22 resource-rich countries (cited by the literature) and 9% reported in a study of 102 countries. Supplemental Table S1 shows the studies used as sources for effectiveness range. 

### 2.7. Calibration and Validation of the Model

We applied the International Society for Pharmacoeconomics and Outcomes Research criteria for model development and reporting to calibrate the model in each country; we compared the disease-specific mortality rates predicted by the model with the national statistics for 16 conditions (excluding COPD mortality, which is universally agreed to be underestimated in national statistics) [46]. Sex- and age-specific model outputs were compared to the source rates and deviations from the expected values were analyzed. Predicted rates within 10% of the references were considered acceptable. In case of greater deviation, risk equations were modified until the parameter was in the acceptable range. Goodness of fit was additionally assessed by plotting predicted versus observed values outcomes, fitting a linear curve through the points with the intercept set at zero, and obtaining a squared linear correlation coefficient. We externally validated the model, checking the results against results of other epidemiological and clinical studies not used for equation estimation or model development. 

## 3. Results

### 3.1. Data to Populate the Model

We identified all the epidemiological and cost parameters needed to populate the model and show the main results of input parameters in Table 1. The rapid review on the effectiveness of ban of advertising and sponsorship showed that smoking prevalence could be reduced by 1% for a partial ban and 9% for a comprehensive ban. 

### 3.2. Current Policies in the Seven Countries

Peru and Mexico are currently at the minimum level of implementation of a ban on advertising and promotion of tobacco products. At the intermediate level of implementation of the ban, which covers national television, radio, or print media, as well as some, but not all, direct and/or indirect forms of advertising are Argentina, Bolivia, and Chile. Finally, at the maximum level of implementation with a comprehensive ban of all forms of advertising, but with varying levels of compliance, are Brazil and Colombia. 

Table 2 shows current policies in the seven countries studied. According to the Report on Tobacco Control in the Americas 2016, Mexico prohibits direct advertising on national radio and television, on billboards, and advertisements from abroad. As for indirect advertising, the ban prohibits product promotion, but allows advertising and display at POS. In Mexico, compliance with the guidelines of Articles 13 and 15 of the WHO FCTC is required to fully comply with the recommendations of the FCTC and to improve the effectiveness of this intervention. Finally, Peru has a ban that does not cover national television, radio, or print media; the MPOWER report score for direct advertising is 9 and does not apply to indirect advertising (available from www.iecs.org.ar/tabaco, access date 30 March 2021) [26,27,28,29,30,31]. According to the categorization in the 2015 MPOWER report, in Argentina, Bolivia, and Chile, the ban covers national television, radio, or print media, as well as some, but not all, direct and/or indirect forms of advertising. The report assigns the following scores on a scale of 0 to 10. Argentina: 9 for direct advertising and 6 for indirect advertising; Bolivia: 9 for direct advertising and 3 for indirect advertising; Chile: 6 for direct advertising and 10 for indirect advertising. In the case of Brazil and Colombia, the ban covers all forms of direct and indirect advertising, with the following scores. Brazil with a score of 9 for both direct and indirect advertising; Colombia: 10 for direct advertising and 5 for indirect advertising.

### 3.3. Health and Economic Effects of Current Strategies Implementation in the Seven Countries 

The policies in advertising that are currently in place in Argentina, Bolivia, Brazil, Chile, and Colombia are already producing health benefits and saving health costs to their systems as a result of their potential to avert a total of 50,000 deaths over the next 10 years. Specifically, over the next 10 years, 199,000 cardiac diseases, 47,000 cerebrovascular diseases, 92,912 COPD cases, and 26,000 cases of cancer could be averted; also, over 1.6 million years of life could be added. Averted events could represent savings totaling I$7.2 billion over the same period. With the largest population in the group of studied countries, Brazil could avert 40,000 deaths, and generate I$5.8 billion in savings (see Table 3). Of note, Mexico and Peru have limited or no bans now.

### 3.4. Additional Potential Benefits of Strengthening Bans of Advertising and Sponsorship

With no ban or a limited ban of tobacco advertising and sponsorship, if Mexico and Peru advanced from their current category to a partial ban, in the next 10 years, Mexico would avert 1700 deaths, almost 10,000 events, would add 47,000 years lived, and save I$313.2 million, whereas Peru would avert 588 deaths, 2300 events, add 14,400 years lived, and save I$60.2 million in health costs. However, if these countries moved to a full ban and implementation, over the next 10 years, Mexico would avert an additional 15,300 deaths, 87,441 events, would add 419,000 years lived, and save US$2.8 billion, whereas Peru would avert 5300 deaths, 20,400 events, add 130,000 years lived, and save I$541.4 million in health costs. Of note, these results are incremental and should be added to the benefits obtained in the first step, with a partial implementation. 

In a second group are Argentina, Bolivia, and Chile, which have bans in intermediate level, if these three countries increased compliance to 100% of existing bans, in the next 10 years, the three countries would avert an additional 759 deaths, 3200 events, would add 19,000 years lived, and save I$111.4 million. Brazil and Colombia already have comprehensive bans with a 90% and 75% compliance, respectively. If they increased compliance to 100%, they would avert an additional 8300 deaths, 55,000 events, would add 239,000 healthy years lived, and save I$1.07 billion in health costs. Of note, these benefits are added to the benefits obtained as a result of the status-quo restrictions.

In a scenario in which all seven countries implemented a comprehensive ban with a 100% compliance, the total numbers over 10 years would be: 98,000 premature deaths, 648,000 events, a total of 3 million healthy years of life would be added, and a total of almost I$15.7 billion in direct healthcare expenses of diseases attributable to smoking would be saved (Table 3). 

## 4. Discussion

Our findings show that the benefits of policies already implemented in Argentina, Bolivia, Brazil, Chile, and Colombia could avert 50,108 deaths, add 1.6 million healthy life years, and save I$7.2 billion, and that optimized ban tobacco advertising and sponsorship in the seven countries, including Mexico and Peru—which have no bans at the moment—could avert more 98,000 premature deaths, and 649,000 events, adding 3 million healthy life years over ten years. This would translate into significant savings for the health system across the Latin American region, with total healthcare savings over I$15 billion. 

The smoking epidemic is well recognized in Latin America; changes are urgently needed to continue reducing mortality due to smoking-related diseases [47]. Estimates suggest that during the last decade, the impact of tobacco was equivalent to 2 to 6 years of losses in life expectancy for males who smoke tobacco at 50 years [47]. In the region, the tobacco industry has been offering a strong opposition and, as a result, passing control legislation has proved difficult [48]. Uruguay has been presented as an example in its efforts to reduce tobacco consumption, followed by Brazil and Panama; in the rest of the region, however, results are varied [48]. 

In Latin American countries there exists an inverse relationship between income level and smoking prevalence, showing that tobacco inflicts greater harm on the most disadvantaged groups [24]. Moreover, it has been suggested that a ban on tobacco advertising could have greater impact on deprived groups, with lower education levels, who are more enticed by advertising [16]. Effective restrictions on advertising are needed in the region; however, bans need to be comprehensive to achieve a reduction in tobacco consumption [4]. Compared to other tobacco control policies, the definition, implementation, and enforcement of tobacco advertising bans is more difficult because of the ever-changing nature of tobacco promotion [49]. These initiatives also require continuous monitoring of, and response to, industry efforts to circumvent bans through new ways to promote tobacco [49,50]. Although the industry contests the bans of advertising, often these result in a higher market concentration benefiting transnational tobacco companies [49,50]. 

Levy et al. showed the significant potential of adopting the highest level of MPOWER tobacco control measures between 2007 and 2014, with an estimated 22 million smoking-associated deaths averted [51]. The impact of any tobacco control intervention depends on the extent of the already implemented control policies or whether several policies are implemented simultaneously [5,52]. In fact, a ban on advertising should be used in the context of other measures. In other studies, our group estimated the health and cost effects of applying tax increase, plain packaging, and smoke-free strategies in the region and found that the first strategy had the most significant effect [22,25,44,45]. However, as it occurs with the other measures, the effects of a comprehensive ban on tobacco advertising and sponsorship would result in important health benefits and savings for the health systems in the region. 

Policies should also include POS marketing, one of the few remaining channels to promote tobacco in many countries [12,53]. As governments implement regulations in traditional media outlets, tobacco companies increasingly market their products to customers at the POS, with product placement, brand exhibition, and price discounts. It has been shown that smoking prevalence decreased as the exposure to a POS display ban increased, with a reduction of 5% and 9% in the male and female populations, respectively [53]. A study in Canada showed that smokers—those most likely to oppose regulations—supported banning POS display of tobacco products, and this should help persuade policymakers to take action [54]. 

Our study has limitations that should be acknowledged. The main limitation is the scarcity of quality evidence on the effectiveness of policies. We estimated direct medical costs related to smoking, a part of total financial burden of tobacco, but not indirect costs, such as productivity losses. The model did not include some conditions related to exposure to tobacco such as breast cancer, diabetes, liver cancer, or kidney failure. In addition, we did not incorporate the use of alternative nicotine delivery products. Another limitation to be considered was that it was not always possible to include high-quality epidemiological information to populate the model. In addition, due to scarcity of information, changes in demographic, economic, and healthcare system characteristics over time were not included in the model. However, our findings offer a robust estimate of financial burden of smoking in seven countries of Latin America, with the best available sources of information in each country, applying a uniform and replicable method. 

In conclusion, advertising ban policies currently in place are producing non-negligible health and economic benefits in five of those countries studied. However, the adoption of a comprehensive ban on tobacco advertising is necessary to substantially enhance the health and financial benefits of reducing the smoking epidemic in the region. 

## Figures and Tables

**Table 1:** Main inputs for the simulation model.

Characteristics	Argentina	Bolivia	Brazil	Chile	Colombia	Mexico	Peru
Population (2015)	43,416,755	10,724,705	207,847,528	17,948,141	48,228,704	127,017,224	31,376,670
Smoking prevalence ^1^							
Male	23.4	20.1	18.0	35.2	20.1	19.8	23.5
Female	18.6	17.7	11.3	31.3	9.9	6.4	15.3
**Crude mortality rate (Male/Female per 10,000) ^2^**							
Acute myocardial infarction	46.1/33.1	8.4/5.5	16.0/11.0	8.3/4.9	19.0/13.7	19.9/13.9	74.6/57.3
Other cardiovascular causes	118.7/104.5	0.9/0.5	3.8/2.9	7.4/8.4	2.3/1.7	2.2/3.1	51.8/57.2
Cerebrovascular disease	52.5/43.9	8.4/8.0	8.8/7.9	9.8/9.6	8.5/9.3	8.1/8.1	52.6/50.7
Pneumonia, influenza	104.4/72.4	17.4/15.9	9.1/8.5	4.2/4.0	3.6/3.1	4.0/3.1	221.0/199.0
COPD	4.3/1.9	1.1/1.3	6.6/4.5	3.7/2.8	7.9/5.8	7.5/5.6	33.2/25.3
Lung cancer	15.6/4.6	3.7/3.1	4.3/2.5	3.9/2.2	3.3/1.9	2.5/1.2	13.5/10.4
**Estimated direct health costs of smoking-related conditions in USD millions**							
Acute myocardial infarction	3242	5114	5006	3944	3835	4848.6	2663
Other cardiovascular causes	2432	3835	1881	2702	1534	3190.4	1850
Annual cardiovascular follow-up	1283	2024	409	1444	34,795	1240.6	1171
Cerebrovascular disease ^3^	4294	5232	4304	4431	2174	4119.1	5058
Pneumonia/influenza	217	276	361	235	325	1309.9	174
COPD ^4^	4394	3969	4824	6133	3463	9236.2	4363
Lung cancer ^5^	17,392	8862	12,279	21,727	10,499	13,792.6	14,081
Mouth cancer ^5^	12,523	6381	9602	15,644	7560	9930.6	9251
Esophageal cancer	14,610	7444	12,161	18,251	8820	11,585.7	11,828
Stomach cancer ^5^	14,262	7267	15,074	17,816	8610	11,309.9	11,546
Pancreatic cancer ^5^	11,827	6026	11,616	14,774	7140	9378.9	9575
Kidney cancer ^5^	12,523	6381	4632	15,644	7560	9930.6	10,138
Tax revenue on smoking ^6^	1926.2	21.5	9511	1346.5	174	2237.4	73.5
GDP (2015) ^6^	583,168.6	33,197	1,774,725	240,215.7	292,080.1	1,144,331.3	192,083.7
GDP per capita (2015) ^6^	13,432	3095	8539	13,384	6056	9009	6122
Price elasticity of demand	−0.299	−0.85	−0.48	−0.45	−0.780	−0.45	−0.7
Total health expenditure (%GDP)	4.8	6.3	8.3	7.8	7.2	6.3	5.5

Abbreviations: GDP, gross domestic product; COPD, chronic obstructive pulmonary disease. Key: ^1^ Population ≥ 35 years expressed in millions; ^2^ Mortality rate per 10,000 people; ^3^ Values include first and following years; ^4^ COPD mild, moderate, and serious included; ^5^ Treatment costs of following years are included; ^6^ In millions of US dollars; exchange rate as mean in December 2015 according to central banks in each country.

**Table 2:** Current advertising ban level and estimated effectiveness for included countries.

Characteristic	Country						
	Argentina	Bolivia	Brazil	Chile	Colombia	Mexico	Peru
Current ban level	Partial	Partial	Comprehensive	Partial	Comprehensive	Absent/Limited	Absent/Limited
Compliance, %	75	55	90	80	75	Non-applicable	Non-applicable
Estimated effectiveness—Status Quo, %(absolute prevalence reduction)	0.75	0.55	8.10	0.80	6.75	-	-

**Table 3:** Estimated cumulative 10-year benefits to ban tobacco advertising.

Country	Advertising Ban	Number of Cases Averted					Life-Years Gained	Costs Averted International Dollars
		Death	MI	Stroke	COPD	Cancer		
Argentina	Status quo (partial ban, 75% compliance)	1378	2183	791	1979	637	33,127	182,534,000
	Step 1: Increase compliance to 100% *	459	728	264	660	212	11,042	60,903,000
	Step 2: Comprehensive ban with 100% compliance *	14,883	23,571	8539	21,371	6876	257,767	1,966,285,000
Bolivia	Status quo (partial ban, 55% compliance)	141	82	155	224	41	3611	23,210,000
	Step 1: Increase compliance to 100%	116	67	127	184	33	2971	18,883,000
	Step 2: Comprehensive ban with 100% compliance	2071	1206	2279	3300	599	53,185	339,496,000
Brazil	Status quo (comprehensive ban, 90% compliance)	40,063	171,265	33,873	76,322	21,729	1375,769	5,830,459,000
	Step 1: Increase compliance to 100%	5569	20,707	4095	9228	2627	166,336	689,054,000
Chile	Status quo (partial ban, 80% compliance)	729	917	853	1658	294	19,083	125,663,000
	Step 1: Increase compliance to 100%	184	231	215	418	74	4809	31,613,000
	Step 2: Comprehensive ban with 100% compliance	7381	9286	8639	16,785	2978	193,212	1,272,435,000
Colombia	Status quo (comprehensive ban, 75% compliance)	7797	24,605	10,898	12,729	3012	203,141	1,045,827,000
	Step 1: Increase compliance to 100%	2787	8795	3896	4550	1077	72,615	377,631,000
Mexico	Step 1: Partial ban with 100% compliance	1705	4403	1072	3600	641	46,564	313,186,000
	Step 2: Comprehensive ban with 100% compliance	15,343	39,626	9645	32,403	5767	419,075	2,781,093,000
Peru	Step 1: Partial ban with 100% compliance	588	357	553	1144	212	14,433	60,151,000
	Step 2: Comprehensive ban with 100% compliance	5294	3210	4974	10,293	1911	129,896	541,355,000
**Total Status Quo**		**50,108**	**199,052**	**46,570**	**92,912**	**25,713**	**1,634,731**	**7,207,693,000**
**Total Comprehensive ban with 100% compliance**		**98,132**	**311,239**	**90,868**	**196,848**	**48,720**	**3,006,636**	**15,659,778,000**

Key: International dollar rates 2018: Argentine peso: 14.09, Brazilian real: 2.77, Chilean peso: 2.2, Colombian peso: 412.36, Mexican peso: 1328.53, Peruvian sol: 1.74. Note: * results are incremental, i.e., numbers in step 1 should be added to status quo, and then numbers of step 2 should be added to the previous two.

## Data Availability

Data supporting results can be found at IECS website (www.iecs.org.ar/tabaco, access date 30 March 2021).

## Supplementary Materials

The following are available online at https://www.mdpi.com/article/10.3390/ijerph18105078/s1, Table S1: Studies used as sources for effectiveness range.
