# Supplementary material for: Optimizing Tobacco Advertising Bans in Seven Latin American Countries: Microsimulation Modeling of Health and Financial Impact to Inform Evidence-Based Policy
Source: Int J Environ Res Public Health. 2021 May 11;18(10):5078. doi: 10.3390/ijerph18105078 (PMC8151006; doi:10.3390/ijerph18105078)
Supplement: Supplementary file 1 [file ijerph-18-05078-s001.zip › ijerph-1164672-supplementary.pdf]

International Journal of
Environmental Research and Public Health

MDPI

Supplemental Material

Table S1. Studies used as sources for effectiveness range.

Source	Number of countries	Intervention	Lower range	Central estimation	Upper range	Estimation type
MPOWER [4] Detected effect	22	Complete	-	7.4	-	Relative reduction of per capita consumption
MPOWER [4]- Detected effect	22	Comprehensive	-	-	-	Relative reduction of per capita consumption
MPOWER [4]- Detected effect	22	Limited-None	0	0	0	Relative reduction of per capita consumption
Tobacco Atlas [51]	NA	Complete	2.5	5	7.5	Relative reduction of per capita consumption
Tobacco Atlas [51]	NA	Comprehensive	1.5	3	4.5	Relative reduction of per capita consumption
Tobacco Atlas [51]	NA	Limited-None	0.0	0	0	Relative reduction of per capita consumption
Tobacco Free Kids [4] Predicted effect for new legislation	22	Complete	-	6.3	-	Relative reduction of per capita consumption
Tobacco Free Kids [4]- Predicted effect for new legislation	22	Comprehensive	-	0	-	Relative reduction of per capita consumption
Tobacco Free Kids [4]- Predicted effect for new legislation	22	Limited-None	-	-	-	Relative reduction of per capita consumption
Tobacco Free Kids [4]	102	Complete	-	9	-	Relative reduction of per capita consumption
Tobacco Free Kids [4]	102	Comprehensive	-	1	-	Relative reduction of per capita consumption
Tobacco Free Kids [4]	102	Limited-None	-	0	-	Relative reduction of per capita consumption
Tobacco Free Kids [16]	30 (developing)	Complete	-	23.5	-	Relative reduction of per capita consumption
Tobacco Free Kids [16]	30 (developing)	Comprehensive	-	13.6	-	Relative reduction of per capita consumption
Tobacco Free Kids [16]	30 (developing)	Limited-None	-	0	-	Relative reduction of per capita consumption
